# Green biosynthesis of magnetic iron oxide nanoparticles using *Mentha longifolia* for imatinib mesylate delivery

**DOI:** 10.1049/nbt2.12090

**Published:** 2022-06-30

**Authors:** Bahareh Naeimipour, Elham Moniri, Ali Vaziri Yazdi, Raheleh Safaeijavan, Hossein Faraji

**Affiliations:** ^1^ Department of Chemical Engineering Science and Research Branch Islamic Azad University Tehran Iran; ^2^ Department of Chemistry Varamin (Pishva) Branch Islamic Azad Universit Varamin Iran; ^3^ Department of Biochemistry and Biophysics Varamin (Pishva) Branch Islamic Azad University Varamin Iran; ^4^ Department of Mechanical Engineering University of Birjand Birjand Iran

## Abstract

In this work, the rapid, facile, and eco‐friendly green process was introduced in the preparation of *β*‐cyclodextrin/magnetic iron oxide nanoparticles by using the aqueous *Mentha longifolia* extracts of *Mentha longifolia*. The obtained nanoparticles were characterised by Fourier transform infrared spectroscopy, x‐ray powder diffraction, field emission scanning electron microscope, and thermogravimetric analysis. Also, effective factors on the synthesis of magnetic nanocomposites including temperature, concentration of the *Mentha longifolia* extract, and concentration of FeSO_4_ solution were optimised by Taguchi design. Moreover, important effective parameters on the adsorption efficiency; such as adsorbent dosage, pH, contact time, and temperature were investigated. The prepared magnetic nanocomposite was applied as a nanocarrier for imatinib mesylate delivery. *In vitro* studies confirmed imatinib mesylate release over 6 h. The nanocarrier showed pH‐dependent imatinib mesylate release with higher drug release at simulated cancer fluid (pH = 5.6) compared to neural fluid (pH = 7.4). Moreover, the sorption isotherms and kinetics for the magnetic nanocomposite were fitted into Langmuir and pseudo‐second order models, respectively. Based on the thermodynamic results, the adsorption of imatinib mesylate onto the nanoadsorbent was found to be spontaneous and exothermic.

## 1 INTRODUCTION

Cancer is a disease with high mortality rate, high incidence rate, and has extremely exposed human health. The considerable adverse effects recorded by chemotherapeutic drugs usually due to their lack of selectivity for cancer tissues and cells, non‐specific targeting, short blood half‐life, and elimination by the immune system often lead to the failure of chemotherapy. To remove these adverse effects, nanocarriers can be used as drug carriers in drug delivery systems [1, 2]. The use of nanotechnology for targeted delivery of drugs has shown considerable prospective in enhancing medicine safety and diminishing medicine relevant toxicity [3].

Magnetic nanoparticles (MNPs) can be used in the field of medical [4, 5], environmental [6, 7, 8], and chemical engineering [9, 10]. Recently, MNPs as drug delivery systems have attracted an enormous attention. Among them, iron oxide‐based MNPs can be effectively utilised for controlled drug‐delivery applications because of its biocompatibility, magnetic properties for selective targeting, and low toxicity [11]. Drug molecules can be conjugated to the shell of MNPs to be injected into the human body and be concentrated in a local tissue due to the effect of an external magnetic field. Owing to the MNPs’ large surface‐to‐volume ratio, it suggests several chemically active sites for drug conjugation [12]. Different methods have been developed to synthesise metal and metal oxide NPs either physically or chemically. These techniques are toxic, expensive, and have high energy requirements [13].

Thus, some efforts have been applied to develop green procedures for the synthesis of NPs to eliminate the disadvantages of previous techniques. The green synthesis of metal and metal oxide NPs using plant extracts is a good alternative technique [14]. Karthick et al. synthesized the gold nanoparticles (AuNPs) by using medicinally valued *Adhatoda vasica Nees* [15]. Shankar et al. synthesised thepure metallic silver, AuNPs, and bimetallic Au/Ag nanoparticles by using *Neem (Azadirachta indica*) leaf broth [16].

Green synthesised MNPs further play a significant role for the delivery of drugs, gens or therapeutic agents, and display several advantages over conventional chemical‐based drug delivery systems. This green technique is simple, eco‐friendly nature, and cost effective [17].

In recent years, there have been few published studies on the green synthesis of NPs using plant extracts. Ahmadi and co‐workers described green synthesis of MNPs using *Satureja hortensis* essential oil. The synthesised MNPs were explored for *in vitro* anticancer drug delivery [18]. Sathishkumar et al. successfully synthesised the MNPs using *Couroupita guianensis Aubl* fruit extract for antibacterial and cytotoxicity activities [19].

The *Mentha longifolia* L. (*Mentha longifolia*), usually known as wild mint or horsemint, is an aromatic and medicinal herb which belongs to Labiataes family. The major constituents of this plant include flavonoids, polyphenols, terpenes, carbohydrates, cinnamates, ceramides, and etc [20, 21, 22]. During the last decade, extracts of plants such as *Moringa oleifera*, *Aspalathus linearise*, *Tabernaemontana divaricata green*, *Aegle marmelos leaves* and *Hibiscus rosa sinensis* have been used in the synthesis NPs of NiO, NiFe_2_O_4_, ZnSnO_3_, ZnFe_2_O_4_, NiO, and ZnO, respectively [23, 24, 25].

Cyclodextrins (CDs) are cyclic oligosaccharides consisting of alpha (six‐membered), beta (seven‐membered), and gamma (eight‐membered sugar ring molecules) or more glucopyranose units joined by *α*‐(1→4) linkage. The advantage of CDs in parenteral formulation includes stabilisation of drugs unstable in an aqueous environment, reduction of drug irritation at the site of administration, solubilisation of drug, and so forth. CDs, cyclic oligosaccharides, have been used for targeting drug delivery due to a distinctive structure, inherent biocompatibility and amphiphilicity. All the groups of drugs are not appropriate substrates for CDs complexation. Drug molecules to be complexed with CDs should have certain characteristics described below. Melting point temperature of the substance is below 250°C; more than five atoms (C, P, S, and N) form the skeleton of the drug molecule; molecular weight is between 100 and 40 *g mol* ^*−1*^; and solubility in water is less than 10 *mg ml* ^*−1*^ [26, 27, 28].

Among the CDs, *β*‐cyclodextrins (beta‐CD; *β*‐CD) have been the most widely used for delivering several kinds of drugs. *β*‐CD is a non‐toxic cyclic oligosaccharide with a molecular structure having a hydrophobic internal cavity [29]. *β*‐CD nanostructures make them appropriate for numerous applications in food [30], agriculture [31], and pharmaceutical [32] industries. *β*‐CD is ideal for DDS due to efficient drug complexation and loading, perfect cavity size, relatively low cost, and availability [33].

Imatinib mesylate (IM; C_29_H_31_N_7_O_CH_3_SO_3_H), the mesylate salt of imatinib, is the first targeted anticancer drug to be clinically confirmed. Imatinib mesylate is one of the most commonly used anticancer drugs for the treatment of chronic myeloid leukaemia and acute lymphocytic leukaemia [34, 35].

Experimental design is an effective technique to reduce the number of experiments as well as the cost of experimentation.

Various design of experiments methods have been proposed to enhance the efficiency of synthesis processes such as Taguchi, Box–Behnken, central composite design, D‐optimal etc. Response surface methodology as a statistical technique, is useful to study the influence of the individual parameters and their possible interaction besides the optimization of the condition, with the minimized error of the experiments and least number of experiments. Also, the use of the Taguchi orthogonal array would obviously minimize the number of experimental runs. It is important to analyse all parameters simultaneously using a few tests [36, 37, 38].

The plant‐based biological method is a deliberated ideal method due to high reproducibility, low cost, less reaction time, eco‐friendliness, and elimination of the cell culture step. Plant extracts contain various kinds of phytochemicals that serve as stabilizing agents and are strong reducing, which drive the synthesis of nanoparticles. Thus, the shape, size, and other properties may vary depending on the source and nature of the plant being used [39]. The novelty of this research focusses on the utilization of these plant extract. The main advantage of using extracts is that they are the mild, renewable and non‐toxic reducing and stabilizing agents, eliminating the need for expensive polymeric capping agents and stabilizers. In comparison with the previous work, few studies have presented on the synthesis of Fe_3_O_4_ nanoparticles from *Mentha longifolia*. Also, iron is a cost‐effective alternative compared with other expensive metals. In this paper, magnetic Fe_3_O_4_ nanoparticles were synthesized using *Mentha longifolia* leaf extract. In addition, Fe_3_O_4_ NPs/3‐(glycidoxypropyl) trimethoxysilane (GPTMS) were coated with *β*‐CD and compared to unmodified Fe_3_O_4_. The as synthesized *β*‐CD @ Fe_3_O_4_ NPs/GPTMS were characterized with analytical techniques such as FT‐IR, FE‐SEM/EDX, XRD and thermo gravimetric analyser (TGA). The mechanisms of the release of IM from the *β*‐CD @ Fe_3_O_4_ NPs/GPTMS in various environment; namely simulated human blood and cancer fluids were studied.

## 2 EXPERIMENTAL SECTION

### 2.1 Reagents and chemicals

Standard IM was bought from Arastoo pharmaceutical company (Iran, Tehran). GPTMS (C_9_H_20_O_5_Si), iron (II) sulphate heptahydrate (FeSO_4_.7H_2_O), *β*‐CD (C_42_H_70_O_35_), sodium dihydrogen phosphate monohydrate (NaH2PO4) and disodium hydrogen phosphate (Na2HPO4) were purchased from Merck Co (Darmstadt, Germany). All of the other chemical reagents were of analytical grade and were obtained from Merck. Ultrapure deionized water (Milli‐Q) was used throughout the work. In all experiments, the stock standard solutions of IM (500 *mg L* ^*−1*^) were prepared in deionised water.

### 2.2 Instruments

The morphological characteristics of nanoparticles were characterised using a field emission scanning electron microscope (FE‐SEM, KYKY‐EM3200, China) at the voltage of 10 *kV*. Fourier transform infrared spectra of the nanoparticles were acquired using a Thermo Nicolet IR100 FTIR instrument (Waltham, Mas‐sachusetts, USA) in the range of 400–4000 *cm* ^*−1*^ in KBr discs. X‐ray diffraction (XRD) patterns of nanoparticles were identified using X‐ray diffractometer (STOE‐STADV, Germany). The thermal behaviour of nanoparticles was carried out using a thermogravimetric analyser (TGA‐TA, Q600, USA). UV–Vis spectrophotometry was evaluated by using a UV‐2100 spectrophotometer (Shimadzu, Japan).

### 2.3 Plant material and extract preparation

The leaves of *Mentha longifolia* plant were bought from local market (Iran, Tehran). The leaves of *Mentha longifolia* were washed several times using deionised water, dried at room temperature, and powdered using an electrical mill (Basic Analytical Mill, Germany). For *Mentha longifolia* extract, 2 *g* of chopped plant was added to 100 *ml* of deionised water and then decanted for 20 min at high temperature. The plant extract was filtered with filter paper (No.1) and Whatman filter paper (No.2), respectively. The plant extract was preserved in the refrigerator at 4°C until use. Preparation of *Mentha longifolia* extracts (1 and 3 *g*) was carried out according to the same procedures.

### 2.4 Experimental design for synthesis of Fe_3_O_4_ nanoparticles (Fe_3_O_4_ NP_s_)

The synthesis of Fe_3_O_4_ NPs were designed by applying the Taguchi experimental design to predict the optimised preparation conditions. Here, three selected parameters, including the temperature (*°C*), concentration of the extracted *Mentha longifolia* (*%*), and concentration of FeSO_4_ solution (*M*) were used. As can be seen in Table 1, the temperature was within 25°C–70°C, the concentration of extracted *Mentha longifolia* was varied from 1*%* to 5*%*, and the concentration of FeSO_4_ was varied from 0.1 to 5 *M*. This design requires nine runs with three parameters at three levels. Numerical optimization for the synthesis of Fe_3_O_4_ NPs was carried out using Minitab software (Minitab^®^16.1.1).

**TABLE 1:** The studied factors and their levels in the Taguchi design

	Factor	Level 1	Level 2	Level 3
A	FeSO_4_ concentration (mol L^−1^)	0.1	0.5	1
B	Plant extract concentration (%)	1	3	5
C	Temperature (°C)	25	50	70

### 2.5 Synthesis of Fe_3_O_4_ NPs

Initially, FeSO_4_ solutions at three concentrations, 0.1, 0.5, and 1 *mol L* ^*−1*^ were prepared by dissolving 2.78, 13.9, and 27.8 *g* in 100 *ml* of deionised water, respectively. 15 *ml* of the extracted *Mentha longifolia* was added drop‐wise to the above solutions. Complete reduction of the iron ions was performed by stirring for 24 *h* at 25°C–70°C. The colour of the solution converted from yellow to brown which indicated formation of Fe_3_O_4_ NPs. The solutions were centrifuged at 1000 *rpm* for 5 *min*, washed with deionised water, and finally dried for 12 *h*.

### 2.6 Synthesis of Fe_3_O_4_ NP_s_ modified with GPTMS (Fe_3_O_4_ NP_s_/GPTMS)

In brief, 0.5 *g* of Fe_3_O_4_ NPs, 2.5 *ml* of GPTMS, and 47.5 *ml* of toluene were added to 100 *ml* of the volumetric flask. The mixture was stirred at gentle reflux at 95°C. After 48 *h*, the solution was centrifuged at 10,000 *rpm* for 10 *min*. After that, 30 *ml* of toluene was added into the above solution drop‐wise under stirring for 15 *min*. Then, the above solution was immediately centrifuged at 10,000 *rpm* for 10 *min*.

### 2.7 Synthesis of *β*‐CD‐coated Fe_3_O_4_ NP_s_/GPTMS (*β*‐CD@Fe_3_O_4_ NP_s_/GPTMS)

**FIGURE 1:**
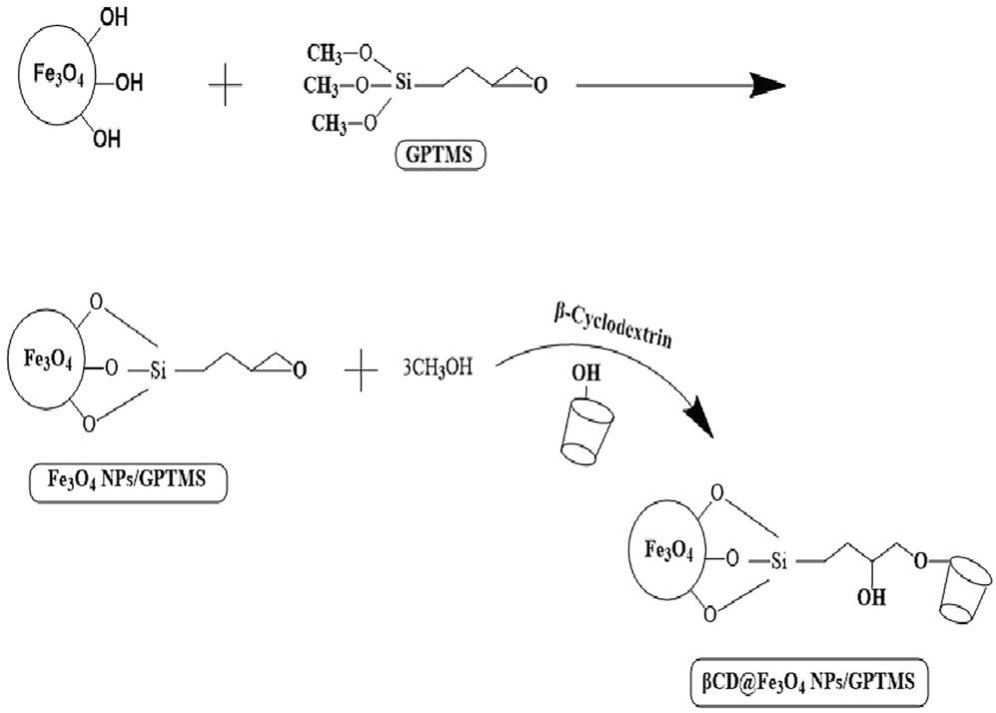
Schematic diagram of the preparation of *β*‐CD @Fe_3_O_4_ NP_s_/(glycidoxypropyl) trimethoxysilane (GPTMS)

For the synthesis of Fe_3_O_4_ NPs/GPTMS modified with *β*‐CD, 0.5 *g* of Fe_3_O_4_ NP_s_/GPTMS and 0.5 *g* of *β*‐CD were added in the round‐bottom flask after the addition of acetate buffer and refluxed for 48 *h* at 45°C. Next, the precipitates of *β*‐CD@Fe_3_O_4_ NPs/GPTMS were washed with acetate buffer and deionised water two times sequentially, and dried in an oven (Memmert, Germany) at 40°C for 24 *h*. The schematic diagram of *β*‐CD @Fe_3_O_4_ NP_s_/GPTMS is presented in Figure 1.

### 2.8 Adsorption of IM using *β*‐CD @Fe_3_O_4_ NP_s_/GPTMS nanoadsorbent

All the batch adsorption tests were carried out on a rotary shaker (KS 4000i control, Germany) with a speed of 250 *rpm*. Typically, 0.01 *g* of *β*‐CD @Fe_3_O_4_ NP_s_/GPTMS and 20 *ml* of IM solution were mixed at pH = 5 for 15 *min*. After that, the solution was centrifuged at 6000 *rpm* for 15 *min*. Next, the supernatant was filtered through a 0.22 *μm* syringe filter (Millipore, Bedford, MA). Finally, the amount of drug concentration in supernatant was analysed using the UV‐Vis spectrophotometer at 256 *nm* in triplicate. The adsorption capacity of IM was calculated by following equation:

(1) $$q_{e}=\left(C_{\text{0}}{‐C}_{e}\right)\times V/M$$

Here, q_*e*_ (*mg g* ^*−1*^) denotes the adsorption capacity, C_*e*_ (*mg L* ^*−1*^) denotes the equilibrium concentration, C_0_ (*mg L* ^*−1*^) denotes the initial concentration of IM in solution, V (*L*) denotes the volume of solution, and *M* (*g*) denotes the amount of *β*‐CD @Fe_3_O_4_NPs/GPTMS.

### 2.9 Loading and *in vitro* release of IM onto the *β*‐CD @Fe_3_O_4_ NP_s_/GPTMS

Loading of *β*‐CD@Fe_3_O_4_NPs/GPTMS with IM was investigated as follows: Firstly, 0.2 *g* of the *β*‐CD @Fe_3_O_4_ NP_s_/GPTMS was added to 50 *ml* of IM solution at 25°C and stirred for 1 *h* at 270 *rpm* using a magnetic stirrer. The precipitate was collected from the solution, and the amount of loaded IM in the *β*‐CD@Fe_3_O_4_ NP_s_/GPTMS was measured at 256 *nm*.

*In vitro* drug release from *β*‐CD @Fe_3_O_4_ NP_s_/GPTMS was conducted at 37°C on a stirrer at 270 *rpm* in a phosphate buffer saline (PBS; 50 *ml*) with the pH value of 7.4 and 5.6. At certain time points, 3 *ml* of solution was collected and 3 ml of fresh PBS was added. The amount of the collected medium was analysed using UV–Vis spectrophotometry at 256 *nm*.

### 2.10 Isotherm, kinetic, and thermodynamic experiments

Adsorption isotherm experiments were studied by adding 0.01 *g* of *β*‐CD @Fe_3_O_4_ NP_s_/GPTMS to 20 *ml* solutions containing a different initial IM concentration of 2–100 *mg L* ^*−1*^, and the solutions were shaken for 1 *h*. Four adsorption isotherms, such as Langmuir [40], Freundlich [41], Temkin [42], and Dubinin‐Radushkevich (D‐R) [43] models, were used to describe the equilibrium adsorption of IM from the aqueous solution.

(2) $$\text{Langmuir}\;\text{model:}\;C_{e}{\text{/q}}_{e}{\;=\;1\text{/K}}_{L}q_{\text{max}}\;+\;C_{e}{\text{/q}}_{\text{max}}$$

(3) $$\text{Freundlich}\;\text{model:}\;\text{Ln}\;q_{e}\;=\;1\text{/n}\;\text{Ln}\;C_{e}\;+\;\text{Ln}\;K_{F}$$

(4) $$\text{Temkin}\;\text{model:}\;q_{e}=\text{RT/b}\;{\text{LnC}}_{\text{e}}+\text{RT/b}\;\text{LnA}$$

(5) $$D‐R\;\text{model:}\;\text{Ln}\;q_{e}\;=\;\text{Ln}\;q_{s}\;‒\;K_{\text{DR}}\varepsilon^{2}$$

In which, q_*e*_ (*mg g* ^*−1*^) and *q*_max_ (*mg g* ^*−1*^) were the capacity of IM adsorbed per gram onto *β*‐CD @Fe_3_O_4_ NP_s_/GPTMS at equilibrium and the maximum IM sorption capacity corresponding to complete monolayer coverage onto *β*‐CD @Fe_3_O_4_ NP_s_/GPTMS, respectively. C_*e*_ (*mg L* ^*−1*^) was the equilibrium IM concentration in the aqueous solution. K_*L*_ (*L mg* ^*−1*^), K_*F*_ (*mg g* ^*−1*^ *) (L mg* ^*−1*^ *)* ^*1/n*^, and K_DR_ (*mol* ^*2*^ *kJ* ^*−2*^) were model constants related to the Langmuir, Freundlich, and D‐R isotherm models, respectively. The parameter of ‘n’ was related to the adsorption intensity. R was represented by the ideal gas constant (8.314 *J mol* ^*−1*^ *K* ^*−1*^); *T* was the temperature (*K*); b was constant associated to the heat of IM sorption (*J mol* ^*−1*^), and A was the Temkin constant. Also, the parameter of Ɛ was related to the Polanyi potential.

For kinetic studies, the synthesised nanoadsorbent with a 20 *mg L* ^*−1*^ solution of IM was performed at different contact times. On the other hand, the influence of temperature on the adsorption of IM by *β*‐CD @Fe_3_O_4_NPs/GPTMS was studied with 20 *mg L* ^*−1*^ of IM at different temperatures (298–323 *K)*. The solution was centrifuged, and the residual IM concentration was analysed using UV‐Vis spectrophotometer. Also, three kinetic models (pseudo‐first order (PFO) [44], pseudo‐second order (PSO) [45], and intra‐particle diffusion (IPD) [46] models were tested for studying the adsorption mechanism.

(6) $$\text{PFO}\;\text{model:}\;\text{Log}\;\left(q_{e}-\;q_{t}\right)\;=\;\text{Log}\;q_{e}–{\text{K}}_{1}q_{t}/2.303$$

(7) $$\text{PSO}\;{\text{model:t/q}}_{t}{\;=\;1\text{/K}}_{2}{q_{e}}^{2}{\;+\;\text{t/q}}_{e}$$

(8) $$\text{IPD}\;{\text{model:q}}_{t}{\;=\;K}_{i}t^{1/2}{\;+\;C}_{i}$$

Here, q_*t*_ (*mg g* ^*−1*^) was the adsorption capacity at time *t*; the parameter of ‘*t*’ was the adsorption time of IM on *β*‐CD @Fe_3_O_4_ NP_s_/GPTMS; and C_*i*_ was the thickness of the boundary layer of the ID kinetic model. K_1_ (*min* ^*−1*^), K_2_ (*g mg* ^*−1*^ *min* ^*−1*^), and K_*i*_ (*g mg* ^*−1*^ *min* ^*−0*.*5*^) were model constants associated to the PFO, PSO and, IPD kinetic models, respectively.

The enthalpy change (ΔH^o^; *J mol* ^*−1*^), the entropy change (ΔS^o^; *J mol* ^*−1*^ *K* ^*−1*^), and the Gibbs‐free energy (ΔG^o^; *kJ mol* ^*−1*^) were calculated to describe the influence of rising temperatures on the adsorption of IM onto *β*‐CD @Fe_3_O_4_ NP_s_/GPTMS. The thermodynamic parameters for the adsorption procedure can be evaluated from the relationship of adsorption isotherms and temperature. Thermodynamic parameters are obtained from the following equations:

(9) ΔG°=−RTLnKc

(10) LnKc=ΔS°/R−ΔH°/RT

(11) Kc=mqe/Ce

In the above equation, R (*J mol* ^*−1*^ *K* ^*−1*^ *)*, *T* (*K*), and *m* (*g*) are the ideal gas constant, the temperature, and the adsorbent dose. Also, K_*c*_ (*L mol* ^*−1*^) represents the equilibrium constant which denotes the ratio of equilibrium concentration of IM adsorbed onto the nanoadsorbent. Upon the above equations, the curve of Ln K_*c*_ versus 1/T provides a straight line and from the intercept and slope of the line, Δ*S*° and Δ*H*° were calculated.

## 3 RESULTS AND DISCUSSION

### 3.1 Taguchi method

In this study, the synthesis of Fe_3_O_4_ NPs was designed by applying the Taguchi OA method to predict the optimised preparation conditions. The experiments were conducted based on the L_9_ orthogonal array (three variables, three levels, and nine experimental runs). All the tests were performed in triplicates and mean values of response were reported. Experimental conditions and the results of the size and morphology of the nanoparticles were shown in Table 2. At first, the size of biosynthesised nanoparticles was chosen as a response. The FE‐SEM images showed that all the nanoparticles from all experiments have an average diameter of 14–23 *nm*, which means no significant change in size occurred from different conditions of experiments. After that, the response was changed to the morphology of nanoparticles and they were divided into 3 groups (good, medium, and bad) which are illustrated in Table 2. The FE‐SEM images of Fe_3_O_4_NPs are shown in Figure S1a‐i. Figure S1f, h, *i* shows the FE‐SEM images of Fe_3_O_4_NPs, which are spherical with a smooth surface (good; 3000). On the other hand, Figure S1a, d, g displays the FE‐SEM images of Fe_3_O_4_NPs with some deformation in apparent and high agglomeration in morphology, due to the sticking effect of MNPs (medium; 2000). Also, images (Figure S1b–e) show the FE‐SEM images of biosynthesised Fe_3_O_4_NPs with hexagonal structure (bad; 1000).

**TABLE 2:** Experimental results: A factor denotes the first variable (FeSO_4_ concentration), B factor denotes the second variable (plant extract concentration), and C factor denotes the third variable (temperature)

Exp.No	A	B	C	Morphology	Mean diameter NP (nm)	StDev (*m* ^*3*^ *s* ^*−1*^)
1	1	1	25	2000	16.40	5.77
2	1	3	50	1000	16.43	4.90
3	1	5	70	1000	14.69	4.64
4	0.5	1	50	2000	15.57	5.89
5	0.5	3	70	1000	20.24	9.23
6	0.5	5	25	3000	18.97	6.95
7	0.1	1	70	2000	18.53	6.06
8	0.1	3	25	3000	23.37	7.61
9	0.1	5	50	3000	22.28	7.73

#### 3.1.1 Main effect plot

In this study, the effects of FeSO_4_ concentration, plant extract concentration, and temperature on the particle size at three different levels (1, 2 and 3) were studied. The main effect plot of the nanoparticles’ size is displayed in Figure S2. The main effect plot was used to show the relationship between the factors and their response in the form of the morphology of the nanoparticles. It was observed that by transferring the concentration of FeSO_4_ from level 1 (1 *M*) to level 3 (0.1 *M*), the nanoparticle morphology improved. In other words, with decreasing FeSO_4_ concentration, the particle morphology is more favourable. The results indicate that changing this parameter is effective on the response variable. Besides, the temperature is a significant factor on the response. As temperature rises from level 1 (25°C) to level 3 (70°C), the morphological quality of the nanoparticles was reduced.

#### 3.1.2 Contour plots

**FIGURE 2:**
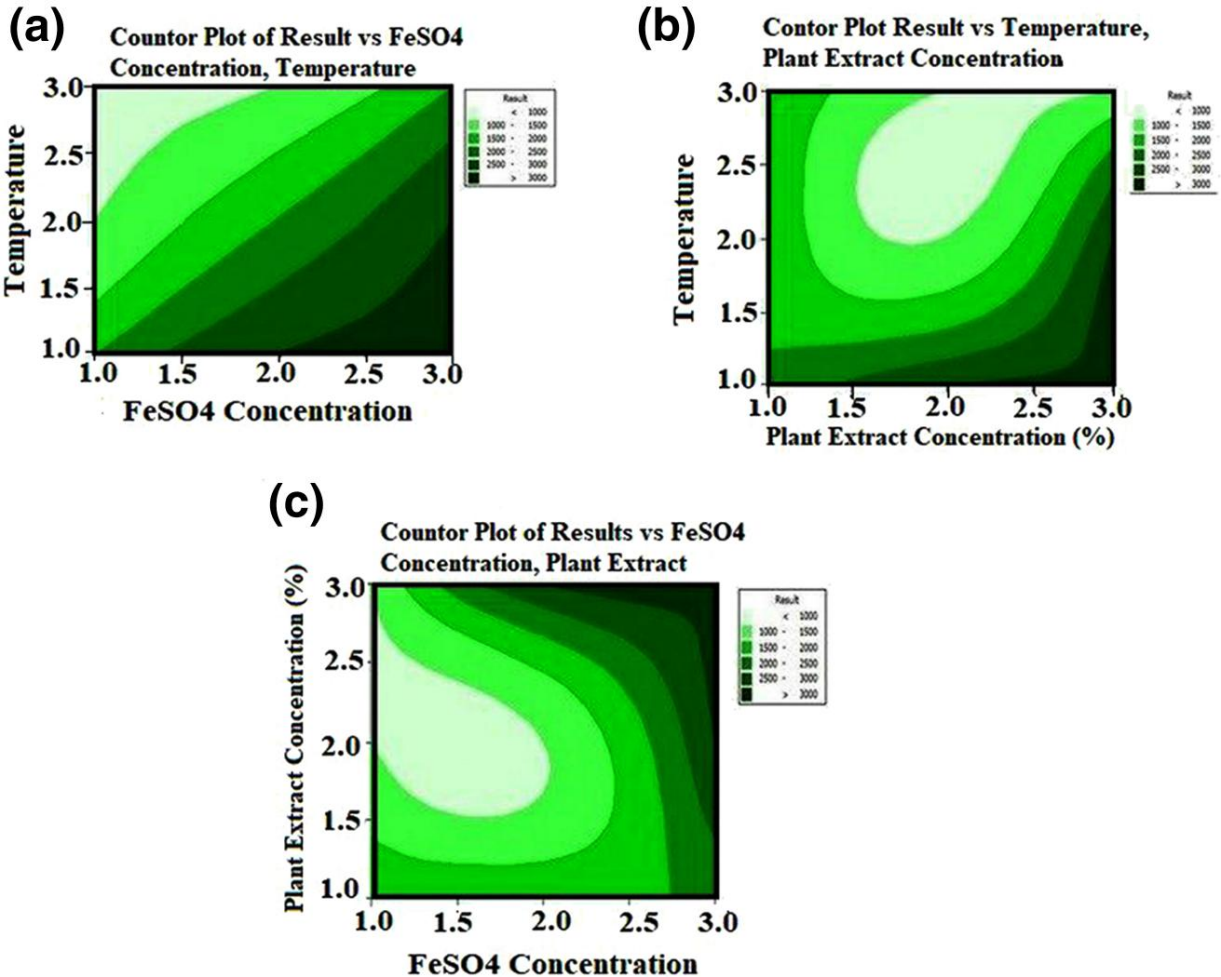
Contour plots for the mutual effects of (a) FeSO_4_ concentration and temperature; (b) temperature and plant extract concentration; and (c) FeSO_4_ concentration and plant extract concentration on the nanoparticle size of synthesised Fe_3_O_4_ NPs

In this study, the effects of FeSO_4_ concentration, plant extract concentration, and temperature on the morphology of nanoparticles were investigated at three different levels, and the obtained results are expressed as contour plots. These contour plots analysed by Taguchi design to determine the optimal conditions and the simultaneous effect of two factors on the morphology of Fe_3_O_4_ NPs and the obtained results are shown in Figure 2. Figure 2a displays the 2‐D contour plot of FeSO_4_ concentration and temperature against the morphology quality of nanoparticles. As can be seen, the quality of nanoparticles morphology was reduced by increasing the temperature from level 1 (25°C) to level 3 (70°C), and decreasing the FeSO_4_ concentration from level 3 (0.1 *M*) to level 1 (1 *M*). Figure 2b shows the simultaneous effect of temperature and plant extract concentration on the response. As observed, the best response was obtained in the highest plant extract concentration (level 3; 5*%*) and lowest temperature (level 1; 25°C). Figure 2c indicated the contour plot of FeSO_4_ concentration and plant extract concentration on response. As shown, the morphology of the nanoparticles improves with increasing concentration of FeSO_4_ from level 1 (0.1 *M*) to level 3 (1 *M*) as well as increasing concentration of plant extract from level 1 (1*%*) to level 3 (5*%*).

#### 3.1.3 Regression analysis

In this paper, only effective variables such as FeSO_4_ NPs concentration, plant extract concentration, and temperature were analysed. The least square linear regression was used. Regression analysis was investigated to develop the relationship between the parameters. The related equation is described as

Result = 1667 + 667 FeSO_4_ concentration +167 plant extract concentration %−667 Temperature.

Optimal conditions according to this plot were level 1 for temperature, level 3 for FeSO_4_ concentration, and level 3 for plant extract concentration.

### 3.2 Optimization of parameters

#### 3.2.1 Effects of adsorbent dosage and contact time

The effects of nanoadsorbent dosage and contact time on IM sorption at pH = 5 and 25°C are shown in Figure S3. In these tests, the amount of *β*‐CD @Fe_3_O_4_ NP_s_/GPTMS at a range of 0.005–0.015 *g* was investigated. By increasing *β*‐CD @Fe_3_O_4_ NP_s_/GPTMS dosage and contact time, the removal percentage of IM was increased. The removal efficiency for IM at equilibrium time was estimated to be 30*%* using 0.015 *g* of the nanoadsorbent. The adsorption capacity of *β*‐CD @Fe_3_O_4_ NP_s_/GPTMS for IM increased very fast within 30 *min*, slightly after 30 min and the removal of IM was almost constant. On the other hand, by increasing *β*‐CD @Fe_3_O_4_ NP_s_/GPTMS dosage and contact time, the adsorption capacity of IM was decreased.

#### 3.2.2 Effects of temperature

Figure S4 shows the adsorption capacity of the IM solutions under pH = 5 at different temperatures. As shown in Figure S4, adsorption capacity of IM increases as the temperature decreases. Also, the adsorption capacity increased when the amount of initial concentration raised from 1 to 100 *mg L* ^*−1*^, and the maximum adsorption capacity of IM was about 45% (*T* = 25°C). Additionally, increasing the initial IM concentration from 1 to 100 *mg L* ^*−1*^ at 298 *K* has shown the increase in the adsorption efficiency from 2% to 46*%*.

#### 3.2.3 Effect of contact time

The effect of contact time of IM onto the *β*‐CD @Fe_3_O_4_ NP_s_/GPTMS at different contact time (2, 5, 10, 20, 30, 45, 60, 90 and 120 *min*) are shown in Figure S5. The maximum adsorption efficiency of 100*%* was observed at the contact time of 30 min while the other factors were temperature 25°C, pH = 5, and adsorbent dosage 0.015 *g*. It can be observed that the adsorption efficiency of *β*‐CD @Fe_3_O_4_ NP_s_/GPTMS for IM was significantly increased during the first 30 *min*, after which it remained almost constant. Rapid adsorption efficiency of IM at the initial time of the adsorption procedure could be related to the active sites on the *β*‐CD @Fe_3_O_4_ NP_s_/GPTMS surfaces.

#### 3.2.4 Effect of pH

**FIGURE 3:**
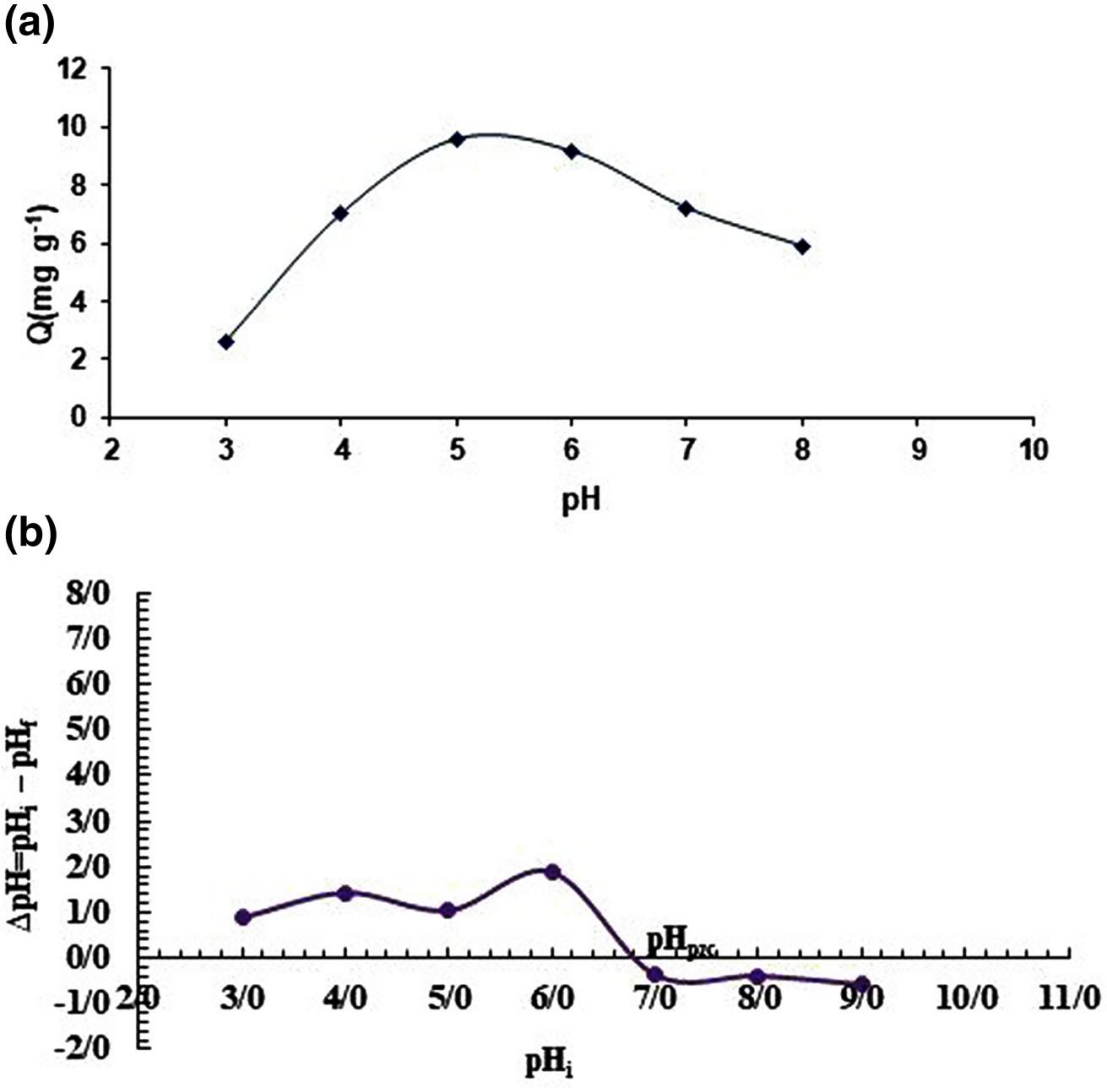
The effect of pH (a) and pH PZC (b) on the adsorption of Imatinib mesylate (IM) using *β*‐CD @Fe3O4 NPs/(glycidoxypropyl) trimethoxysilane (GPTMS) (Experimental conditions; initial concentration, 20 mg L^−1^; nanoadsorbent dosage, 0.015 g; contact time, 30 min; and temperature = 25°C)

For investigating the influence of the pH value on the *β*‐CD @Fe_3_O_4_ NPs/GPTMS adsorption efficiency, the pH of solutions were tested in the range of 3–8. Based on the results (Figure 3a), by increasing the solution pH from 3 to 5, the adsorption capacity was enhanced, reaching the maximum value of 9.6 *mg g* ^*−1*^ at pH = 5. On the other hand, the decrease in the adsorption capacity at pH higher than 5 could be attributed to the decomposition of *β*‐CD @Fe_3_O_4_ NPs/GPTMS in an alkaline pH. Consequently, pH = 5 was chosen as an optimum pH in the next experiments.

Additionally, to confirm the accuracy of evaluating the optimal pH, the pH _PZC_ was investigated. The results of this study indicated that the zeta potentials of *β*‐CD @Fe_3_O_4_ NPs/GPTMS decreased when the pH increased from 3 to 9. At pH < 6.8, the surfaces of *β*‐CD @Fe_3_O_4_ NPs/GPTMS are positively charged. At pH > 6.8, the zeta potential of *β*‐CD @Fe_3_O_4_ NPs/GPTMS is negative. Thus, the pH _PZC_ value for IM adsorption was 6.8 (Figure 3b).

### 3.3 Characterisation

#### 3.3.1 XRD analysis

The crystalline phase of Fe_3_O_4_ NP_s_ and *β*‐CD @Fe_3_O_4_ NP_s_/GPTMS were identified with XRD analysis (Figure S6). For Fe_3_O_4_ NP_s_ (Figure S6a), diffraction peaks with 2*θ* = 31.1°, 36.8°, 53.1°, and 78.0° appeared, which were attributed to the crystal planes of (220) (311) (422), and (440), respectively. The XRD pattern of *β*‐CD @Fe_3_O_4_ NP_s_/GPTMS (Figure S6b), shows characteristic peaks at 2*θ* = 30.9°, 34.4°, 45.6°, 67.8°, and 78.0° corresponding to the (220) (311) (400) (511), and (440), respectively (ICDD Reference card No: 19‐0629). These results confirmed that Fe_3_O_4_ NP_s_/GPTMS was successfully modified by *β*‐CD.

#### 3.3.2 FT‐IR analysis

**FIGURE 4:**
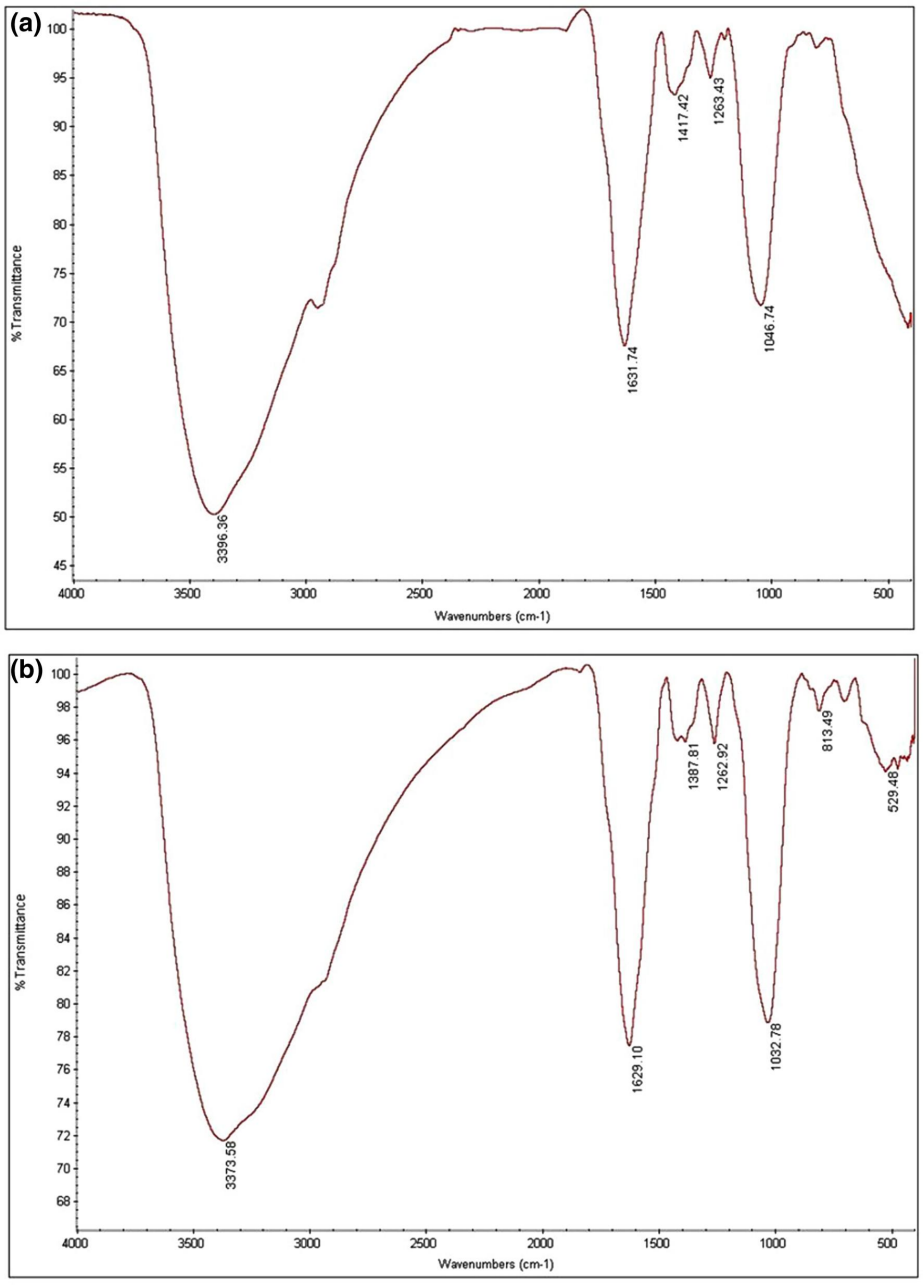
FT‐IR spectra of Fe_3_O_4_ NPs (a) and *β*‐CD @Fe_3_O_4_ NPs/(glycidoxypropyl) trimethoxysilane (GPTMS) (b)

FT‐IR spectra of Fe_3_O_4_ NPs and *β*‐CD @Fe_3_O_4_ NPs/GPTMS were characterised in the range of 400–4000 *cm* ^*−1*^ (Figure 4). As observed in Figure 4a, frequencies observed at 1629 and 3373 *cm* ^*−1*^ were attributed to the stretching vibrations of *C* = *O* and OH groups, respectively. The peaks ranging from 1000 to 1300 *cm* ^*−1*^ were assigned to the C‐O and C‐C stretching vibrations. The resulting peak at 529 *cm* ^*−1*^ in this spectrum indicates the formation of magnetite nanoparticles (Fe_3_O_4_ NPs). In the *β*‐CD @Fe_3_O_4_ NP_s_/GPTMS spectrum, the broad peak at 3396 *cm* ^*−1*^ were related to the stretching vibration of OH. The band at 1046, 1263 and 1417 *cm* ^*−1*^ were ascribed to the C‐O, C‒O‒C, and CH_2_, respectively. In addition, the peaks at 1631 *cm* ^*−1*^ related to the out of plane stretching vibration of the OH group (Figure 4b). The stretching vibration for Fe–O groups of Fe_3_O_4_ particles was observed at 529 *cm* ^*−1*^, which shifted to 498 cm^−1^ after coating with *β*‐CD.

#### 3.3.3 TGA analysis

Thermogravimetric curves of Fe_3_O_4_ NP_s_ and *β*CD@Fe_3_O_4_ NP_s_/GPTMS were obtained from room temperature to 600*º*C at 10*º*C *min* ^*−1*^. Results of TGA were indicated in Figure S7. Weight loss of 59.03*%* and 68.5*%* were detected for Fe_3_O_4_ NP_s_ and *β*‐CD @Fe_3_O_4_ NP_s_/GPTMS, respectively. Based on thermograms, 9.5*%* weight loss was observed for samples due to the modification of Fe_3_O_4_ NP_s_ with polymer and the decomposition of *β*‐CD. Thermal resistance of the samples occurred at about 400°C.

#### 3.3.4 FE‐SEM analysis

**FIGURE 5:**
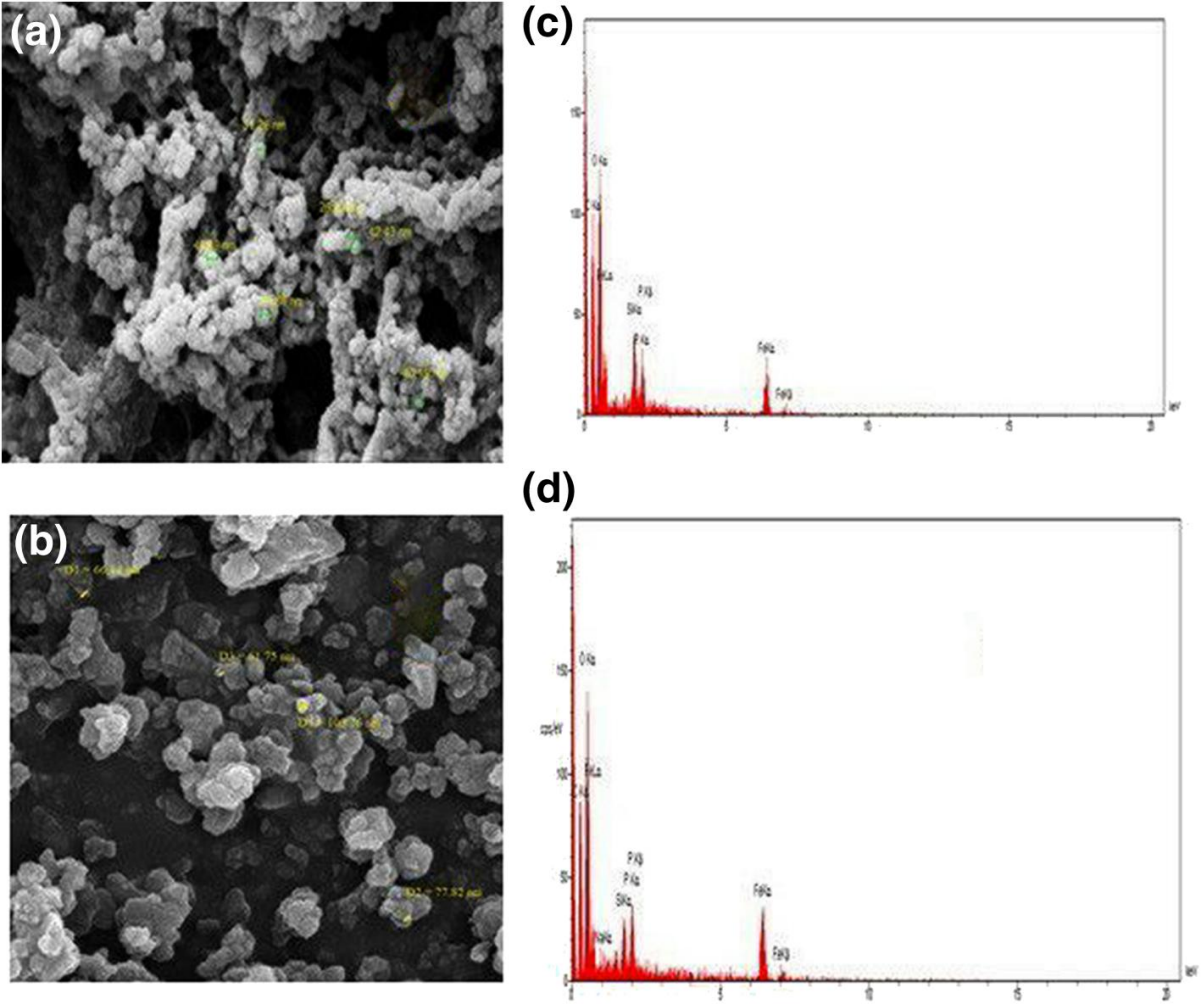
FE‐SEM images of Fe_3_O_4_ NP_s_ (a), *β*‐CD @Fe_3_O_4_ NP_s_/(glycidoxypropyl) trimethoxysilane (GPTMS) (b) and EDX results of Fe_3_O_4_ NP_s_ (c), *β*‐CD @Fe_3_O_4_ NP_s_/GPTMS (d)

FE‐SEM images were investigated to estimate the surface morphology of the Fe_3_O_4_NP_s_ and *β*‐CD @Fe_3_O_4_ NP_s_/GPTMS as shown in Figure 5. As can be seen in Figure 5a, the images indicated that the particles have spherical shape. The average diameter of Fe_3_O_4_ NP_s_ was approximately 38 *nm*. After coating, the FE‐SEM images of *β*‐CD @Fe_3_O_4_ NP_s_/GPTMS indicated that the surface of agglomerated nanoparticles have spherical shapes with particle size of 61–103 *nm* (Figure 5b). Accordingly, the average size of *β*‐CD @Fe_3_O_4_ NP_s_/GPTMS will increase with agglomeration.

Also, Figure 5 shows the EDX elemental mapping images of Fe_3_O_4_ NPs and *β*‐CD @Fe_3_O_4_ NP_s_/GPTMS. As can be seen in Figure 5c, the EDX image of Fe_3_O_4_ NP_s_ indicate the presence of C (35.26 *w %*), Fe (14.53 *w %*), Si (1.91 *w %*), O (44.63 *w* %), Na (0.84 *w* %) and *p* (2.32 *w* %) elements and for *β*‐CD @Fe_3_O_4_ NP_s_/GPTMS, Figure 5d indicates the presence of C (35.35 *w %*), Fe (12.69 w%), Si (1.66 w*%*), O (47.41 w*%*), Na (0.57 *w %*) and *p* (1.66 *w %*) elements which confirm the presence of Fe_3_O_4_ and *β*‐CD nanoparticles on the surface. After surface modification of Fe_3_O_4_ NPs with *β*‐CD, the atomic weight ratio of C and O were increased; however, in contrast, the weight percentage of Fe and Si were decreased. As the results were confirmed, coating of *β*‐CD particles on the Fe_3_O_4_ NP_s_/GPTMS surface was successful.

### 3.4 Investigation of adsorption behaviour of *β*‐CD @Fe_3_O_4_ NPs/GPTMS

#### 3.4.1 Isotherm study

The adsorption isotherms of IM on the *β*‐CD @Fe_3_O_4_ NPs/GPTMS were investigated at various temperatures of 298, 308, and 323 *K*. According to Table 3, the adsorption capacity (*q*_max_) of *β*‐CD @Fe_3_O_4_ NPs/GPTMS for IM decreased with increasing the temperature. The *q*_max_ for IM on *β*‐CD @Fe_3_O_4_ NPs/GPTMS, evaluated from the Langmuir isotherm model, was 62.11 *mg g* ^*−1*^ at 298 *K*. High correlation coefficient (*R* ^2^) values obtained for the Langmuir isotherm model suggest that the Langmuir model was quite fitted to the isotherm data. Table 3 has shown that the *R* ^2^ for the Langmuir isotherm model were 0.9912, 09,946 and 0.9941 at 298, 308, and 323 *K*, respectively. The R_*L*_ value of the *β*‐CD @Fe_3_O_4_ NPs/GPTMS for IM was observed to be 0.21 at 325 *K*. In addition, the value of ‘n’ was estimated to be from 1.55 to 1.84 for the adsorption of IM using *β*‐CD @Fe_3_O_4_ NPs/GPTMS. The ‘n’ value greater than one represents that the adsorption of IM on the nanoadsorbent is undesirable. In the 298–325 *K* range, the b parameter values of the Temkin isotherm model increase with the increase of temperature. In contrast, the *q*_*s*_ values of the D‐R isotherm models decrease with the increase of temperature range from 298–323 *K*.

**TABLE 3:** Equilibrium isotherm parameters for adsorption of Imatinib mesylate (IM) on *β*‐CD @Fe_3_O_4_ NPs/(glycidoxypropyl) trimethoxysilane (GPTMS) (Experimental conditions: nanoadsorbent dosage: 0.015 *g*; contact time: 30 *min*; and pH = 5)

Isotherm model	Parameters	*T* = 298*K*	*T* = 308*K*	*T* = 323*K*
Langmuir	*q*_max_ (*mg g* ^*−1*^)	62.11	48.31	42.55
	K_*L*_ (*L mg* ^*−1*^)	0.04	0.038	0.036
	R_*L*_	0.201	0.207	0.21
	*R* ^2^	0.9912	0.9946	0.9941
Ferundlich	K_*F*_ (*mg g* ^*−1*^ *)*	3.45	2.91	3.2
	*(L mg* ^*−1*^ *)* ^*1/n*^ n	1.55	1.64	1.84
	*R* ^2^	0.9556	0.9805	0.9794
Temkin	A (*L mg* ^*−1*^)	0.48	0.34	0.33
	b (*J mol* ^*−1*^ *)*	193.89	231.87	276.57
	*R* ^2^	0.982	0.9905	0.9903
Dubinin‐radushkevich	q_*s*_ (*mg g* ^*−1*^)	40.87	32.73	28.81
	K_DR_ (*mol* ^*2*^ *kJ* ^*−2*^)	0.00002	0.00002	0.00002
	*R* ^2^	0.9561	0.8528	0.8504

#### 3.4.2 Kinetic study

The kinetic study for the adsorption of IM onto *β*‐CD @Fe_3_O_4_ NP_s_/GPTMS at various adsorbent dosage (i.e., 0.005, 0.01, and 0.015 *g*) was investigated using three kinetic models, including the PFO, PSO, and IPD. Kinetic parameters of the adsorption process were calculated and listed in Table 4. As shown in Table 4, the adsorption kinetic data in this study could be well explained by the PSO kinetic model. However, the calculated equilibrium constant (*q*_*e*_, cal) for *β*‐CD @Fe_3_O_4_ NP_s_/GPTMS decreased as the adsorbent dosage was increased from 0.005 to 0.015 *g*. Noticeably, the PSO rate constant, k_2_ was increased for IM as the adsorbent dosage of the nanoadsorbent was raised. Furthermore, if the PSO kinetic model provides the best fit to the adsorption process, physisorption mainly controls the adsorption process.

**TABLE 4:** Kinetic parameters for adsorption of Imatinib mesylate (IM) on *β*‐CD @Fe_3_O_4_ NPs/(glycidoxypropyl) trimethoxysilane (GPTMS) (Experimental conditions: initial concentration: 20 *mg L* ^*−1*^; pH = 5; and temperature = 25°C)

Kinetics models	Parameters	0.005	Adsorbent dosage (*g*) 0.01	0.015
PFO	K_1_ (*min* ^*−1*^)	0.11	0.15	0.07
	q_*e*_ (*mg g* ^*−1*^)	8.43	6.26	1.34
	*R* ^2^	0.994	0.9639	0.9994
PSO	q_*e*_ (*mg g* ^*−1*^)	13.99	11.27	8.13
	k_2_ (*g mg* ^*−1*^ *min* ^*−1*^)	0.03	0.07	0.17
	*R* ^2^	0.9996	0.9998	0.9999
IPD	Ki_1_ (*g mg* ^*−1*^ *min* ^*−0*.*5*^)	1.94	1.45	0.24
	Ki_2_ (*g mg* ^*−1*^ *min* ^*−0*.*5*^)	0.5	0.19	0.17
	C_1_ (*mg g* ^*−1*^)	4.59	4.91	6.59
	C_2_ (*mg g* ^*−1*^)	10.44	9.88	6.92
	R_1_^2^	0.9903	0.9987	0.9673
	R_2_^2^	0.9335	0.8126	0.9938

Abbreviations: IPD, intra‐particle diffusion; PFO, pseudo‐first order; PSO, pseudo‐second order.

#### 3.4.3 Thermodynamic study

The influence of temperature on the adsorption of IM by *β*‐CD @Fe_3_O_4_ NP_s_/GPTMS was examined by using 25 *mg L* ^*−1*^ of IM at different temperatures (298–323 *K)*. As can be seen in Table S1, the value of ∆G^o^ decreased from −9.12 to −9.67 with increasing temperature. The negative values of ΔG^o^ confirmed that the adsorption efficiency of IM on *β*‐CD @Fe_3_O_4_ NP_s_/GPTMS was feasible and spontaneous. Meanwhile, the negative value (−2506.01) of ΔH^o^ confirmed the exothermic nature of the adsorption process. Moreover, the positive value of ΔS^o^ confirmed that the degree of freedom of the *β*‐CD @Fe_3_O_4_ NPs/GPTMS increased during the adsorption process. The ΔG^o^ range for physical adsorption is between 0 and −20 *kJ mol* ^*−1*^.

### 3.5 Drug release study

The release behaviour of IM from *β*‐CD @Fe_3_O_4_ NP_s_/GPTMS was studied in simulated human blood fluid (SHF, pH = 7.4) and simulated cancer fluid (SCF, pH = 5.6) as the release fluid. For the first 1 *h*, under SHF, about 12*%* of IM was released from the nanocarrier (Figure S8). However, only approximately 54*%* of drug was released from the nanocarrier after 6 *h*. In contrast, at pH = 5.6, the IM loaded *β*‐CD @Fe_3_O_4_ NP_s_/GPTMS released about 37*%* of IM after 1 *h* and 97*%* after 6 *h*. The nanocarrier in the SCF (pH = 5.6) has shown that the release of IM was faster than that at pH = 7.4. It may due to the OH groups on the surface of *β*‐CD. H‐bonding interaction between the drug and nanocarrier at acidic pH (SCF; pH = 5.6) is weak and drug release increases.

In order to indicate the advantage of the present method, we have compared the obtained results in the drug release of IM over the *β*‐CD @Fe_3_O_4_ NPs/GPTMS with some reported nanocarriers in the viewpoint of drug release. Table 5 displays drug release percentages of various nanocarriers for IM release as noted in previous works [47, 48, 49, 50]. The release of the IM depends on the interaction between the drug molecule and nanocarrier, that is, π–π interaction between *β*‐CD @Fe_3_O_4_ NPs/GPTMS and IM. The comparison data shown that *β*‐CD @Fe_3_O_4_ NPs/GPTMS indicated higher the release percentage compared with other nanocarriers.

**TABLE 5:** Comparison of the release of Imatinib mesylate (IM) drug with various reported nanocarriers

Nanocarrier	Drug release (%)	Time (*h*)	Ref.
FPL‐DOX/IM	72	13	[47]
IM/GNP‐HCIm	30	8	[48]
IM/MNs@p (NVCL‐co‐VAc)‐DABA	80	6	[49]
PBCA nanoparticles	10	48	[50]
β‐CD @Fe_3_O_4_ NPs/GPTMS	97	6	This work

## 4 CONCLUSION

In conclusion, Fe_3_O_4_NP_s_ were produced by an environmentally friendly approach using aqueous extract of *Mentha longifolia* leave as a nanocarrier. Green chemistry is the preferred route for synthesis of metal nanoparticles because of it is non‐noxious, viable, ecological friendly, fast, and cost effective. We reported a suitable method for the attaching of *β*‐CD onto the surface of Fe_3_O_4_ NPs/GPTMS. By using the Taguchi orthogonal array, the effective parameters on the synthesis of Fe_3_O_4_NP_s_ were optimised (pH = 5, temperature = 25^*o*^ *C*, adsorbent dosage = 0.015 *g*, and contact time = 30 *min*). According to the release curve, *β*‐CD @Fe_3_O_4_ NPs/GPTMS released 54% and 97*%* of drug after 6 h in neutral and acidic fluids, respectively. Also, adsorption results were consistent with the Langmuir isotherm model and PSO kinetic model. The positive value of Δ*S*° suggests that the adsorption increased the randomness during the adsorption of IM by *β*‐CD @Fe_3_O_4_ NP_s_/GPTMS. According to the thermodynamic results, the adsorption method was exothermic (Δ*H*°<0) and spontaneous (Δ*G*°<0) in nature.

## CONFLICT OF INTERESTS

The authors declare that they have no known competing financial interests or personal relationships that could have appeared to influence the work reported in this paper.

## PERMISSION TO REPRODUCE MATERIALS FROM OTHER SOURCES

None.

## Supporting information

Supporting Information S1

## Data Availability

Data sharing is not applicable to this article as no new data were created or analysed in this study.
