# Supplementary material for: Green biosynthesis of magnetic iron oxide nanoparticles using Mentha longifolia for imatinib mesylate delivery
Source: IET Nanobiotechnol. 2022 Jun 30;16(6):225–37. doi: 10.1049/nbt2.12090 (PMC9353862; doi:10.1049/nbt2.12090)
Supplement: Supplementary file 1 — Supporting Information S1 [file NBT2-16-225-s001.docx]

**Green Biosynthesis of Magnetic** [**Iron Oxide**](https://www.sciencedirect.com/topics/agricultural-and-biological-sciences/iron-oxides) **Nanoparticles using *Mentha longifolia* for Imatinib Mesylate Delivery**

Bahareh Naeimipour ^1^, Elham Moniri ^2*^, [Ali Vaziri Yazdi](https://www.sciencedirect.com/science/article/abs/pii/S0141813016302318#!) ^1^, Raheleh Safaei Javan ^3*^, Hossein Faraji ^4^

^1^Department of Chemical Engineering, Science and Research Branch, Islamic Azad University, Tehran, Iran

**^2^** Department of Chemistry, Varamin (Pishva) Branch, Islamic Azad Universit, Varami, Iran

**^3^**Department of Biochemistry and Biophysics, Varamin (Pishva) Branch, Islamic Azad University, Varamin, Iran

^4^ Department of Mechanical Engineering, University of Birjand, Birjand, Iran

^*^moniri30003000@yahoo.com

^*^safaeijavan@gmail.com

**Figure Captions:**

**Fig S1.** SEM images of Fe_3_O_4_ NPs by nine experiments (a - i).

**Fig S2.** Main effect plot of Fe_3_O_4_NP_s_ synthesized by extract of *Mentha Longifolia.*

**Fig S3.** The effect of nanoadsorbent dosage and contact time on the (a) removal efficiency of IM (b) adsorption capacity of IM. (Experimental conditions: initial concentration: 20 *mg L^-1^*; pH=5; and temperature = 25 *ºC*)

**Fig S4.** The effect of temperature on the adsorption capacity of IM (Experimental conditions: initial concentration: 20 *mg* *L*^-^*^1^*; nanoadsorbent dosage: 0.015 *g*; contact time: 30 *min*; and pH=5)

**Fig S5.** The effect of contact time on the adsorption of IM using β-CD @Fe_3_O_4_ NPs/GPTMS (Experimental conditions: initial concentration: 20 *mg* *L*^-^*^1^*; nanoadsorbent dosage: 0.015 *g*; pH=5; and temperature = 25 *ºC*)

**Fig S6.** XRD patterns of Fe_3_O_4_ NP_s_ (a) and β-CD @Fe_3_O_4_ NP_s_/GPTMS (b)

**Fig S7.** TGA curves of Fe_3_O_4_ NP_s_ (a) and β-CD @Fe_3_O_4_ NP_s_/GPTMS (b)

**Fig S8.** IM release study from β-CD@Fe_3_O_4_ NP_s_/GPTMS at different pH of 5.6 and 7.4 at 37 *℃*

**Table Captions:**

**Table S1.** Thermodynamic parameters for adsorption of IM on β-CD @Fe_3_O_4_ NPs/GPTMS (Experimental conditions: nanoadsorbent dosage: 0.015 *g*; contact time: 30 *min*; and pH=5)


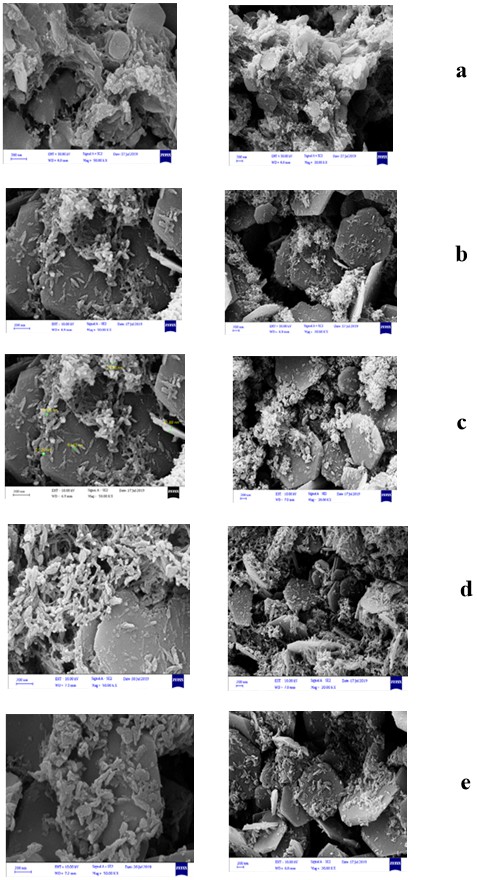


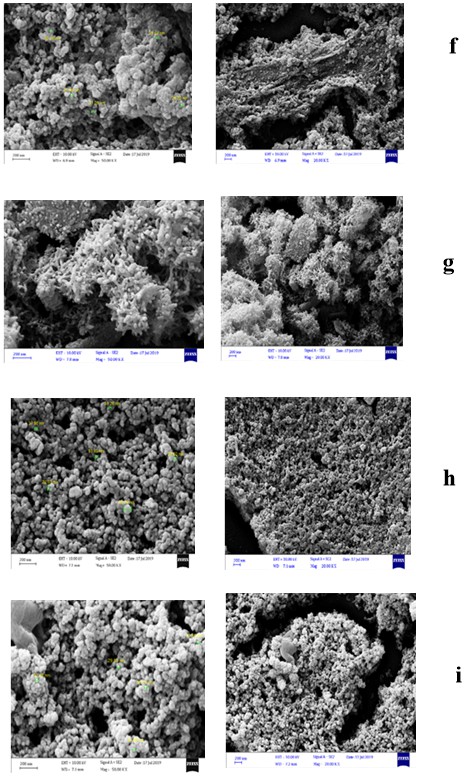


**Fig.S1**


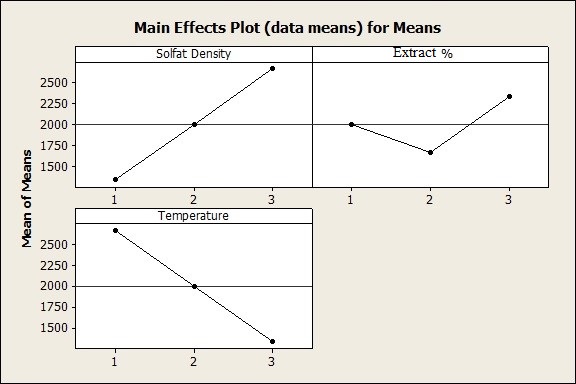


**Fig.S2**

**
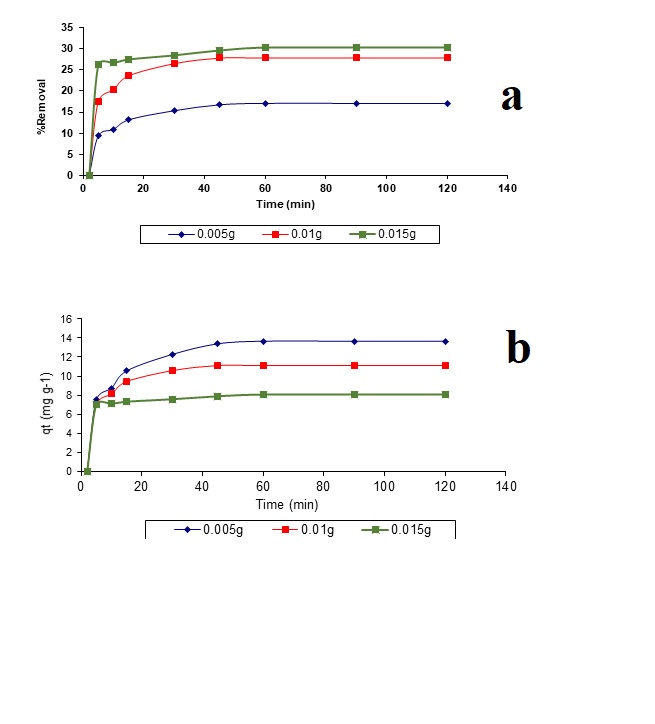
**

**Fig. S3**


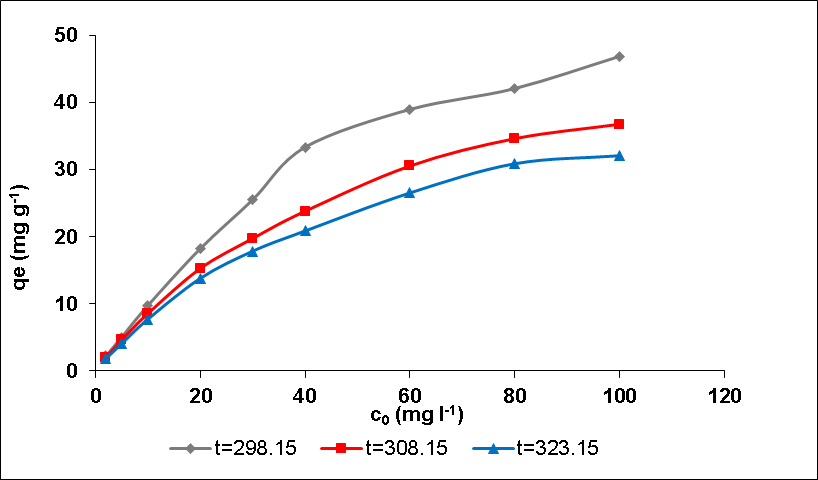


**Fig. S4**


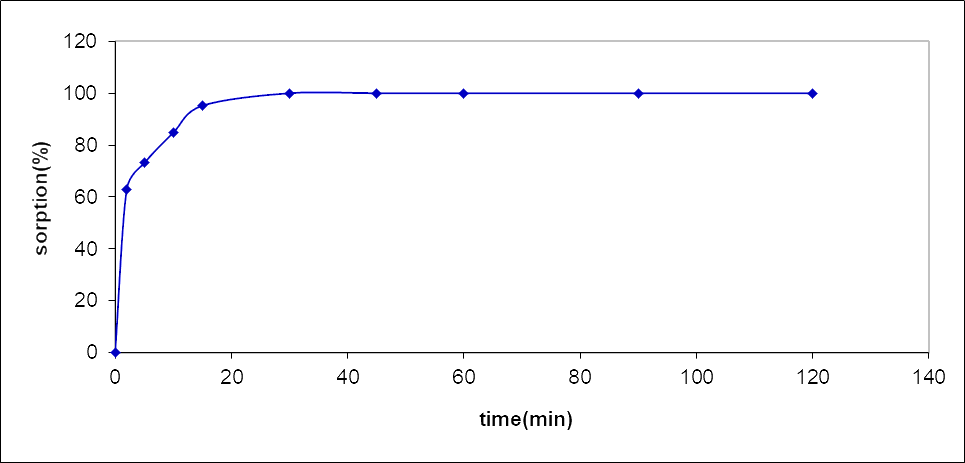


**Fig. S5**


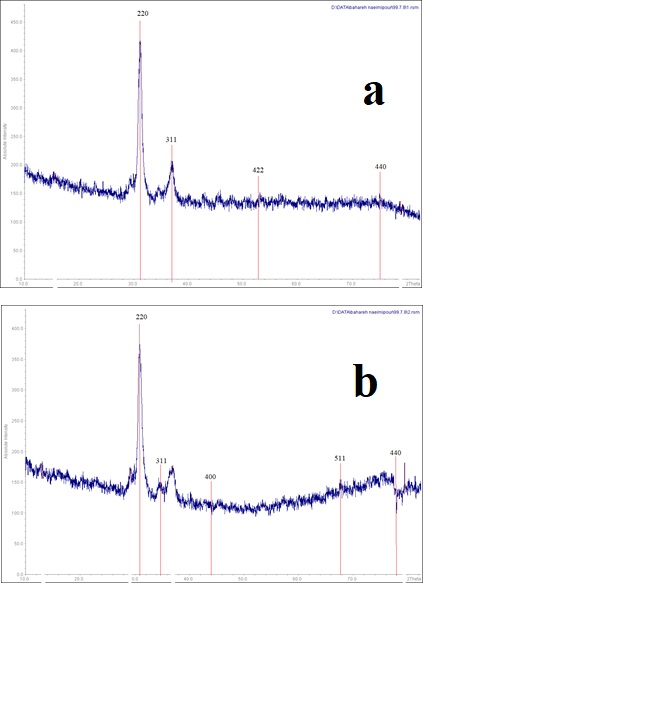


**Fig S6.**


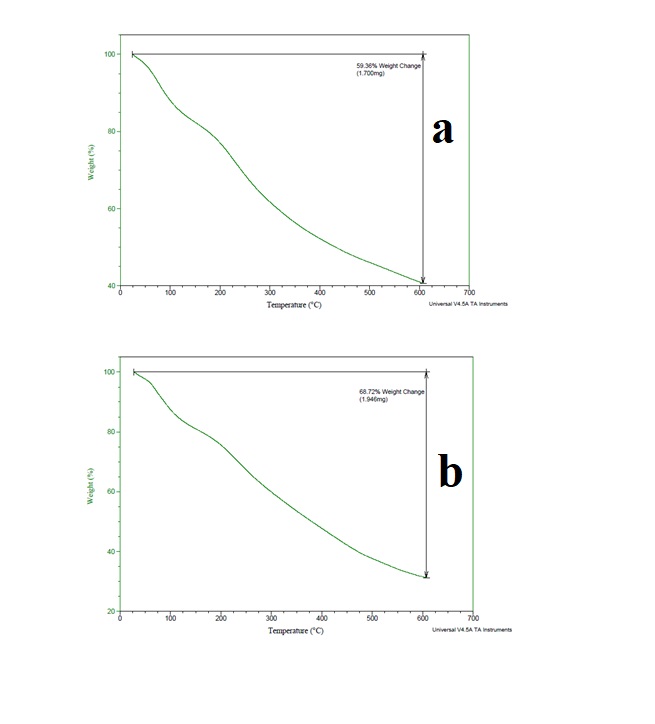


**Fig S7.**


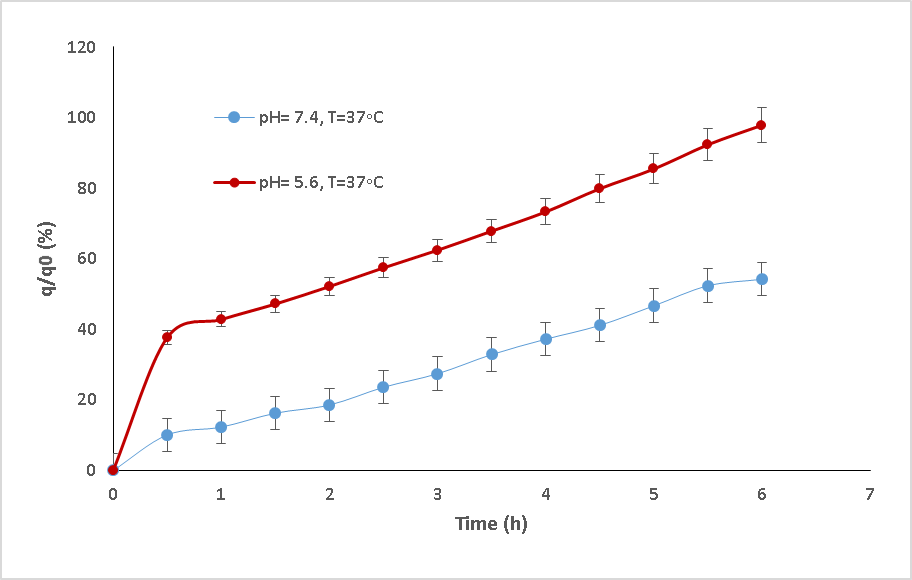


**Fig S8.**

**Table S1.**

| **Δ G^o^ (***KJ mol^-1^***)** | **Δ S^o^ (***J mol^-1^ K^-1^***)** | **ΔH^o^ (***J mol^-1^***)** | **Temperature (***K***)** |
| --- | --- | --- | --- |
| -9.12 | 22.17 | -2506.01 | **298.15** |
| -9.34 |  | | **308.15** |
| -9.67 |  | | **323.15** |
